# Mood Self-Assessment in Children From the Age of 7

**DOI:** 10.5964/ejop.v14i3.1408

**Published:** 2018-08-31

**Authors:** Aurélie Simoës-Perlant, Céline Lemercier, Christelle Pêcher, Sarah Benintendi-Medjaoued

**Affiliations:** a Laboratoire CLLE, Université de Toulouse CNRS, UT2J, Toulouse, France; Department of Psychology, Webster University Geneva, Geneva, Switzerland; University of South Wales, Newport, United Kingdom

**Keywords:** emotion, imagination mood induction, emotional vocabulary, children, development

## Abstract

The evaluation of emotions is one of the main challenges facing theorists and applied psychology researchers. In children, in order to focus on subjective feelings, psychologists mainly use non-verbal scales that measure both the intensity and valence of the emotions felt. The use of these scales poses a main research questions: What is the children’s knowledge of the emotion presented? In order to properly assess the emotional state of a child, it is first necessary to measure the child’s understanding of the major characteristics of emotion. Secondly, it is important to assess the child’s ability to designate the primary emotion associated with a particular situation, and assess how these emotional situations alters their own assessment of their emotional state. This research aims to know if children from the age of seven to eleven can be emotionally induced and if this induction varies in the lifespan.

Vocabulary is the set of words belonging to a language. Among these words, some are the names of day-to-day objects or people (e.g. toy, cup, Richard), others are verbs (e.g. eat, name), and some are adjectives (e.g. black, big). The adjective sub-set includes the emotional adjectives, that is those which add information on the emotional state of the "word" that we want to define (angry, happy). Since the study by Carey (1978; see also Barrett, 1995), it is established that vocabulary is enriched with age, going from 500-600 words at 2 years of age to 14,000 words at 7 (Barrett, 1995; Carey, 1978), allowing individuals to improve the precision of their discourse and add nuance in their reflections. This paper investigates the critical question of the assessment of their own emotions by children, based on an analysis of their knowledge of emotional vocabulary. More precisely, we will detail a step-by-step approach that we used to develop a new verbal scale for the assessment of emotions by children from 7 to 11.

The assessment of emotions is a key issue for theorists and researchers in applied psychology. Emotion is defined as an individual assessment of an emotion-relevant event (e.g. Frijda, 1987; Lazarus, 1991; Scherer, 1984). Thus, the emotion will vary in time and between subjects (Frijda, 2009). Emotions have two main dimensions: Valence and arousal. Valence corresponds to the way people experience a situation (pleasant vs. unpleasant) and arousal corresponds to the degree of activation (calm vs. excited).

The emotional experience involves noticeable adaptive changes on the peripheral physiological system, action tendencies, the motor system, the subjective feelings and cognitive evaluation (Scherer, 2005). This last component involves the changes in mental states related to emotion, and refers to the subject's assessment of his or her own emotional state.

Specific methods and tools have been developed to assess each component of the emotional experience. Focusing on the subjective feelings, psychologists widely use verbal scales to measure both the intensity and the valence of felt emotions. To rate emotions on this type of scale, respondents are implicitly required to: 1) perceive their own feelings, 2) verbalize them with appropriate words, and 3) modulate their answers to express subtle differences. This process could be considered by no means easy for an adult, and therefore far more difficult for young children.

Some studies on babies’ ability to recognize facial expressions showed that they can perceive and recognize the emotional valence of faces, are able to match body images to vocal information at 6.5 months using emotional cues (Zieber, Kangas, Hock, & Bhatt, 2014), and can discriminate between discrete emotions at an early stage if they are habituated to the stimuli (Nelson & Dolgin, 1985). This ability evolves with increasing age (Gosselin, 2005; Thommen, Châtelain, & Rimbert, 2004) as does early competence in controlling the expression of emotions (Josephs, 1994). Moreover, in these studies, emotions are always compared in pairs (e.g. joy vs. fear), with the principal dimension being the distinction of valence. However, we cannot conclude that children are able to recognize the intensity of these different emotions, which would enable them to distinguish between two emotions of the same valence (e.g. sadness vs. anger). Inversely, young children’s self-assessment capacity develops later, as they require more time to apprehend emotional words and subtle differences (e.g. Lewis, 2011; Lewis, Sullivan, Stanger, & Weiss, 1989). The early development of emotion and mental state vocabulary is well documented (e.g., Johnson-Laird & Oatley, 1989; Ortony, Clore, & Foss, 1987) but few studies have focused on this point in elementary school-aged children and adolescents. According to Saarni (2000), at school entry, children develop sufficient specific linguistic competencies to evoke with words their own emotions as well as those of others. It therefore seems relevant to investigate the verbalization of emotions in this young in-learning population.

Until now, assessing emotions with pre-school children has always involved the use of non-verbal pictorial scales. The most widely used scale is the Self-Assessment Manikin (SAM) developed by Lang (1980), and Bradley and Lang (1994). In its original version, three 9-point visual analog scales represent a figure from smiling to frowning, from wide-eyed to relaxed-sleepy and from large to small. Each scale reflects one dimension of emotions: Valence (positive/negative), arousal (high/low) and dominance (total/null). Respondents must indicate on the three scales which figure best reflects their feelings. The validity of the SAM has been attested in many situations including the assessment of the emotional valence of experimental material, and the rating of emotions from childhood to older ages (e.g., Backs, da Silva, & Han, 2005; Bradley & Lang, 1994; Kuppens, Tuerlinckx, Russell, & Feldman Barrett, 2013; Laukkal & Haapala, 2013; Vasa, Carlino, London, & Min, 2006) or from normal to pathological behaviors (e.g. Gouvousis et al., 2010; Hughes & Kendall, 2008; Kotta & Szamosközi, 2012). There is plenty of evidence that using the SAM with children is easy and practical. But due to the theoretical principles underlying the constructibility of the SAM, this pictural scale cannot discriminate emotions other than positive vs. negative, and intense vs. slight. And the completion of data by additional interviews is difficult, indeed impossible, with young children because of their lack of emotional vocabulary.

It is worth noting here that some verbal scales were developed to assess the emotional state of children. For instance, Cuisinier, Sanguin-Bruckert, Bruckert, and Clavel (2010) created a self-assessment questionnaire in French to characterize childrens’ emotional state using terms such as the following: "*inquiet/*worried", "*énervé*/upset", "*joyeux/*joyful", "*triste*/sad", "*fier/*proud", "*mal à l’aise/*ill at ease", "*je m’ennuie/*bored", "*content*/happy". Young participants had to rate each item on eight 5-point scales from 1 ("not at all") to 5 ("extremely"), providing a measure of the valence and arousal of their felt emotion. This scale deserves mention because it is both the very first and the only one that investigates the question of children’s self-assessment of emotional state. However, like every study on this topic, there is no prior appraisal of the knowledge of emotional vocabulary of the participants. It is therefore not possible to rule out the possibility that the participants estimated certain items without understanding their meaning. More clearly, each targeted adjective is associated with a scale. With this mono-item structure, the emotional valence and the intensity can be measured but the knowledge of the emotional vocabulary is not controlled. However, one of the main criticisms made against these verbal scales is about the quality of the emotional items to be rated. None of the listed studies took the precaution of making sure that the emotional items were perfectly understood by the participants. If we can imagine that such an omission has only marginal consequences in adults, the situation is quite different in children from 7 to 11 years old, whose vocabulary is still very limited.

It is also essential to consider the Positive and Negative Affect Schedule (PANAS) developed by Watson, Clark, and Tellegen (1988), which is the best-known verbal scale. Originally, the PANAS was underpinned by a 2x2 dimensional structure: (I) pleasant-unpleasant, (II) arousal-calm, (I’) positive-tired and (II’) negative-relaxed, the second set of dimensions being 45° rotations of the first set in the same factor space. In its original version, the PANAS uses 20 adjectives. Respondents must rate on a 5-point scale how they feel the described emotions at that precise moment. The PANAS is commonly used in specific cultural groups and clinical contexts. As a consequence, this tool was adapted following linguistic and cultural suggestions. For instance, there is a version for the UK (Crawford & Henry, 2004), a Spanish version for the adult population of Cordoba, Argentina (Moriondo, De Palma, Medrano, & Murillo, 2012), one for the African-American Community in the USA (Merz et al., 2013) and one for the Brazilian adult population (de Carvalho et al., 2013).

Of particular interest here, the PANAS was also adapted for children. For example, Damásio, Cerentini Pacico, Poletto, and Koller (2013) validated a Brazilian version for children between the ages of 7 and 16, with only 8 items, the PANAS C-8. Laurent et al. (1999) developed a version for US children from 4^th^ to 8^th^ grades (9-14 years old), the PANAS-C, with 27 items, which was also used with children with anxiety disorders (Hughes & Kendall, 2009). But the consistency of the internal structure of the questionnaires remains unclear. Moreover, responding to 8 emotional items does not reflect the same cognitive demand as rating 27 items, especially for children. Additionally, proposing a longer questionnaire for children involves the possibility of incorporating linguistic subtleties, which may be useful, but also of creating ambiguities in children’s minds and inducing a reduction in the meaningfulness of answers.

Finally, it is worth mentioning another verbal questionnaire, the Brief Mood Introspection Scale (BMIS), developed by Mayer and Gaschke (1988). This questionnaire is based on the findings of Watson, Clark, and Tellegen (1988). The reliability and validity of the psychometrics were confirmed. The BMIS is made up of 16 adjectives enabling four dimensions of assessment: (I) the pleasant-unpleasant scale, (II) the arousal-calm scale, (I’) the positive-tired scale and (II’) the negative-relaxed scale. For each adjective, participants must circle the response on the associated scale that best indicates how they feel at that moment. The 4-point scale ranges from "definitely do not feel" to "definitely feel". The BMIS exists in a French version adapted by Niedenthal and Dalle (2001). However, to our knowledge, the BMIS has not been used with a child population.

A complementary point is that the BMIS is recognized and used for assessing emotional changes after mood induction in applied research (e.g. Corson & Verrier, 2007; Jallais, Gabaude, & Paire-Ficout, 2014). Mayer, Allen, and Beauregard (1995) developed a series of emotional induction procedures to induce four specific emotions, anger, sadness, joy and fear. These procedures combined the presentation of guided imagery vignettes describing emotional situations to be imagined with the presentation of valence-congruent music. From our point of view, this type of method is appropriate for children as it is fun and non-intrusive.

Taken together, the data emphasize the researcher’s difficulties in assessing the emotional state of children with a tool appropriate for their age. Current verbal scales were not initially designed to take their lexical skills into account, and this may explain why they do not always seem to clearly understand the difference between the different items. In addition, existing graphic scales are too general and do not allow a clear labeling of the emotional experience.

This study aims to investigate the question of the assessment of their own emotions by children based on the analysis of their knowledge of emotional vocabulary. From a methodological perspective, this implies designing a new verbal scale dedicated to the assessment of emotions by children from 7 to 11. From a practical perspective, emotion has an impact on many cognitive skills, especially on school learning abilities. Professionals are increasingly taking emotion into account in the school environment. As knowledge acquisition is a core concern, this study provides both theoretical and practical working methods that better correspond to the activities of instructors and learning specialists.

## Experiment 1

In this first experiment, we investigated whether elementary age children are able to detect the valence of emotional words in terms of positive vs. negative, and whether they are capable of labeling the type of emotion underlying the word (i.e., happiness, anger, sadness or fear). Two sorting tasks were proposed for this purpose.

### Method

#### Participants

The participants were 77 schoolchildren. Four grade-levels were recruited from an elementary school in Toulouse (France). The distribution was as follows: 2^nd^ grade (19 children, *M* = 7.89 years, *SD* = 0.24 years, 10 boys and 9 girls), 3^rd^ grade (20 children, *M* = 8.91 years, *SD* = 0.32 years, 8 boys and 12 girls), 4^th^ grade (17 children, *M* = 10.02 years, *SD* = 0.28 years, 14 boys, 3 girls) and 5^th^ grade (21 children, *M* = 10.90 years, *SD* = 0.31 years, 10 boys, 11 girls). Pupils were pre-selected by their teacher on the basis of their language skills. Although all the pupils of a given class participated in the experiment (depending on their parents’ agreement), only those without any major reading and writing difficulties were included in the analyses.

#### Ethical Clearance

We made sure to respect the French "Code of conduct applied to researchers in behavioral sciences" (Caverni, 1998). As all the subjects were minors, we obtained the agreement of each legal representative. Each participant gave their free and informed consent and we made it clear to them that they could leave the scientific process at any time. Our material was designed in such a way as to leave no misunderstanding on any matter at all. In accordance with the French society of psychology criteria concerning *Deontology and Ethics of Research in Psychology* (http://www.sfpsy.org/), the researchers provided the participants and their parents with any additional information they needed to understand the study after the research was completed. The experimenters were concerned that participants experience the test in a positive manner. So, to avoid any negative effects or misunderstandings, individual and collective debriefings were conducted with the participants. We communicated our results to all the participants. Their anonymity was respected and protected throughout the process.

#### Materials and Design

Estimated frequency per million words was verified using the MANULEX database (Lété, Sprenger-Charolles, & Colé, 2004). Three words or expressions related to each of the four primary emotions (Joy, Anger, Sadness and Fear) were selected, *content* (pleased), *heureux* (happy), and *joyeux* (joyful) for the joy category, *en colère* (angry), *énervé* (annoyed), and *furieux* (furious) for the anger category, *chagriné* (grieved), *malheureux* (unhappy), and *triste* (sad) for sadness and *effrayé* (afraid), *apeuré* (scared), and *inquiet* (worried) for fear. Each word was typed in Calibri font, size 20 and printed onto a 3x7 cm card with a white background. Three 15x10 cm colored boxes (one blue, one red and one white) were also used for the sorting tasks.

#### Procedure

Children were tested individually. The child sat behind a table on which three boxes were placed. The cards with words were spread on the table in front of the child. Before the sorting task, the experimenter asked the child to read each word aloud. When the word was not correctly read, the experimenter read the word aloud to ensure its correct pronunciation. For the first sorting task, the experimenter asked the child to put the positive adjectives in the blue box and the negative adjectives in the red box. The white box was reserved for unknown, neutral or ambiguous adjectives. For the second sorting task, the experimenter again took the 12 cards, mixed them and spread them on the table in a different order. The child was again asked to read them and to group the "words that go together". The number of groups was not limited and was decided freely by the child.

### Results

#### Categorization Task 1: Detection of the Emotional Valence

A set of chi-squared analyses was performed on the correct emotional valence (positive vs. negative), sorting for each emotion-related adjective and for each age group (2^nd^ grade, 3^rd^ grade, 4^th^ grade, 5^th^ grade). All the adjectives were correctly sorted at more than 75% (all *p*’s < .05), apart from the 2^nd^ grade children, who classified *apeuré* at the level of chance (47.37%, χ^2^(1, 19) = .05, ns.).

#### Categorization Task 2: Association by Primary Emotion

The second part of the experiment investigated the grouping of all the emotional words as a free sorting task. In this second part, participants had to group the items that went well together. They did not give any label for the category they produced. We were interested in the number of emotional items grouped "correctly" regarding the four primary emotions. We considered that the sorting was done on the basis of the primary emotion when all the items grouped corresponded to this one. For example "*effrayé*" with "*inquiet*" for Fear or "*joyeux*", "*content*" and "*heureux*" for Joy. If an item was left on its own, we did not count a point for it. Similarly, if two items were grouped on the basis of another potential criterion, e.g. "*joyeux*" with "*triste*", we did not count points either.

First, the data on performances on this second categorization task were analyzed through a one-way ANOVA for 4 (Grade) x 2 (Sex). The results showed a significant effect of the Grade (*F*(3, 69) = 5.03, *p* < .01, η^2^_p_ = .18). The percentage of correct sorting of emotions increased linearly with higher grades, and therefore with age (2^nd^ grade = 68.89% [4.39], 3^rd^ grade = 80.9% [4.38], 4^th^ grade = 88.3% [6.09], 5^th^ grade = 91.4% [4.18]). However, there were neither a significant effect of Sex nor an interaction Grade x Sex (*F* < 1, ns.).

A second set of chi-squared analyses was performed on the task of sorting the adjectives into each of the four studied primary emotions ("Joy", "Anger", "Sadness" and "Fear") for each age group (2^nd^ grade, 3^rd^ grade, 4^th^ grade, 5^th^ grade) (see Table 1).

**Table 1:** Sorting Card Results for the Four Primary Emotions From 2^nd^ Grade to 5^th^ Grade

Grade	Joy			Anger			Sadness			Fear		
	Content	Heureux	Joyeux	En colère	Enervé	Furieux	Chagriné	Malheureux	Triste	Effrayé	Inquiet	Apeuré
2^nd^ Grade	94.74*	100.00*	100.00*	84.21*	84.21*	63.16	36.84	47.37	63.16	57.89	47.37	47.37
3^rd^ Grade	100.00*	95.00*	100.00*	85.00*	90.00*	85.00*	60.00	80.00*	75.00*	85.00*	55.00	65.00
4^th^ Grade	100.00*	100.00*	100.00*	100.00*	100.00*	70.59*	76.47*	100.00*	94.12*	100.00*	52.90	82.35*
5^th^ Grade	100.00*	100.00*	100.00*	100.00*	100.00*	90.48*	80.95*	85.71*	90.48*	85.71*	80.95*	85.71*

*Note.* The asterisks refer to "correct" classification performance above random probability.

In 2^nd^ grade, children properly attributed the primary emotion "Joy" for the items *joyeux* (100% "correct" classification), *heureux* (100% "correct" classification), and *content* (χ^2^(1, 19) = 15.21, *p <* .001). The adjectives *en colère* (χ^2^(1, 19) = 8.9, *p* < .01) and *énervé* (χ^2^(1, 19) = 8.9, *p* < .01) were also classified properly in the Anger group, while *furieux* was scored less than random probability (χ^2^(1, 19) = 1.32, ns.). For the two remaining emotions, all the adjectives were scored below random probability (χ^2^’s, ns). We performed a post-hoc set of chi-squared analyses on the adjectives, sorting each of the three couples of negative emotions ("Sadness/Fear"; "Sadness/Anger"; "Fear/Anger") for 2^nd^ grade children. Only *chagriné* was ranked at less than random probability in the "Sadness/Fear" category (χ^2^(1, 19) = 1.32, ns.). For other items, there was no confusion or ambiguity.

Results indicated that children in 3^rd^ grade sorted all the adjectives associated with "Joy" and "Anger" correctly (all *p’s <* .01). *Malheureux* and *triste* were appropriately classified (respectively, χ^2^(1, 20) = 7.2, *p <* .01 and χ^2^(1, 20) = 5, *p* < .003) in the Sadness group, while *chagriné* was not (χ^2^(1, 20) = .80, ns.). *Effrayé* was correctly classified in the Fear group (respectively, χ^2^(1, 20) = 9.8, *p* < .01) while *apeuré* and *inquiet* scored below random probability (respectively, χ^2^(1, 20) = 1.8, ns.; χ^2^(1, 20) = .20, ns.).

Children of the 4^th^ grade correctly classified all the adjectives referring to "Joy", "Anger" and "Sadness" (all *p*’s < .001). For the emotion Fear, only *inquiet* scored below random probability (χ^2^(1, 20) = .06, ns).

Finally, 5^th^ grade children correctly classified all the adjectives in the four primary emotions "Joy", "Anger", "Sadness", and "Fear" (all *p*’s < .001).

#### Discussion

This study aimed to provide information on the familiarity of children in elementary school (2nd to 5th grade) with emotion-related adjectives referring to four basic emotions: Joy, sadness, anger and fear.

The first clear element is that children are perfectly capable of distinguishing between the negative and the positive valences of emotion-related words (more than 75% of correct answers for all grades and all adjectives). Although the Manulex database considers that French children know the word "*apeuré*" (scared), it was not correctly categorized by the younger participants in our study, suggesting in fact that it was not properly understood. Indeed, this adjective is the only one for which the valence was not correctly identified by the 7-year-olds, in the categorization task. In a study by Vasa et al. (2006), emotional and non-emotional words were presented to children from the ages of 9 to 11 who had to rate their valence for positive vs. neutral vs. threat. The results show fairly good consistency in the valence ratings. In their discussion, the authors explained that they first considered younger children from 7 to 8 but that presenting emotion-related verbal stimuli could create confusions in the experimental paradigm due to variations in children’s reading levels. Our study both confirms and refutes this idea. On the one hand, our results showed that even children under 7 can define the emotional valence of words. On the other hand, we observe that knowing the emotional valence of a word does not predict the ability to associate it more specifically with a group of words referring to the same primary emotion.

In the second task of this experiment, children had to label different emotion-related words in terms of four categories: Joy, fear, sadness and anger. The results showed a linear increase of labeling performance with age/grade. In other words, children learn subtle distinctions between emotions and how to define them progressively. Note here the ambiguities with words such as *chagriné* or *inquiet*, which were placed by younger children in both the sadness and fear categories. There was no gender effect.

One explanation is that to identify emotions clearly, they first need to be signified. Words allow subtle variations in the expression of emotional reactions. In parallel with the development of the behavioral component of emotions (gestures, facial expressions, etc.) in a child, the acquisition of an emotional lexicon begins from 18 months (Beeghly, Bretherton, & Mervis, 1986). Some other studies suggest even earlier acquisition of emotion-related words. Smiley and Huttenlocher (1989), for instance, found that native English-speaking children could use words related to fear and joy from 13 months. These types of emotions are expressed twice as much as other emotions at the same age. Nonetheless, until the age of 35 months, emotion-related words are only used to express the child’s own feelings, and not necessarily correctly. Children thus use emotion-related verbal stimuli before being able to understand and identify their meaning correctly. According to Smiley and Huttenlocher (1989), the emotional lexicon increases with age and becomes more accurate. These results are discussed by Maillochon (2008), who found that French children express more joy (28 to 32 months) and anger (33 to 37 months) than fear.

All authors agree that the basis of an emotional lexicon is acquired at the age of around 2/3 years and increases with age and experience, both in terms of accuracy and quantity of terms. This is in line with a series of studies showing that experiencing emotions at school and training children to understand emotions through story-telling and conversation, help them in the identification and labeling of emotions (e.g. Ornaghi, Brockmeier, & Grazziani, 2014; Widen, Pochedly, & Russell, 2015). Von Salisch, Haenel, and Freund (2013) also demonstrated that the understanding of emotions is dependent on the improvement of non-verbal cognitive abilities (e.g. matrices, similarities, etc.). Non-verbal as well as verbal abilities increase with time and experience, and with knowledge acquired from the family, at school and so on. It is therefore no surprise to observe this linear increase in the labeling of emotions in our experiment.

These first results show the need to develop tools and methods suitable for the age of the child. More precisely, as regards rating the emotions, it seems extremely important to adapt self-rating scales to children’s knowledge of the emotional lexicon. Our first study showed that the youngest children could rate the emotional valence of words but had difficulty in labeling the type of emotions. When using the image-based mood induction procedure proposed by Mayer et al. (1995), it is also important to question whether these very young children can or cannot rate the emotional valence as well as determine the emotional label of everyday situations they are reading. It may have a negative bias on the induction and the emotional ratings.

## Experiment 2

The aim of Experiment 2 was to select a set of sentences intensively associated with one of the four primary emotions (anger, joy, sadness, fear) by the children from 7 to 11 that we could use in Experiment 3, so as to induce a specific emotional state in children with material adapted to their daily concerns. One requirement was that all sentences had to be understood and validated by children, whatever their age or grade. The chosen sentences or situations had to be representative of the daily and social activities of children, in which they could easily imagine themselves. The pool of sentences was carefully composed to be comprehensible by anyone. It was a methodological prerequisite for Experiment 3, to ensure the efficiency of the emotional inductions generated by the sentences. Notice here that contrary to the Mayer and Gaschke (1988) procedure, no music was presented with the vignettes.

### Method

#### Participants

The participants were 155 schoolchildren recruited from four grade-levels of an elementary school in Toulouse (France). The distribution was as follows: 2^nd^ grade (31 children, *M* = 7.80 years, *SD* = 0.25 years, 19 boys and 12 girls), 3^rd^ grade (43 children, *M* = 8.87 years, *SD* = 0.34 years, 17 boys and 26 girls), 4^th^ grade (40 children, *M* = 9.92 years, *SD* = 0.40 years, 22 boys, 18 girls) and 5^th^ grade (41 children, *M* = 10.97 years, *SD* = 0.51 years, 21 boys, 20 girls).

#### Ethical Clearance

The same as for Experiment 1.

#### Materials and Design

With reference to the BMIS (Mayer & Gaschke, 1988), forty-two sentences were constructed to induce emotions in children. Each sentence, designed to induce one of the four studied emotions, reflected a situation that children might encounter in their daily lives among family, friends and school environments (*cf*. Table 2). Four lists of sentences were constructed so that each child was presented with 12 sentences. In each list, three sentences referring to each of the four basic emotions (J = Joy, A = Anger, F = Fear, S = Sadness) were selected and were randomly presented, for example "*C’est ton anniversaire et tous tes amis sont venus les bras chargés de superbes cadeaux" ("It’s your birthday and all your friends have come with a lot of amazing presents")*. The sequences were as follows: Lists 1 and 2 = JAFSAJSFASJF; Lists 3 and 4 = FAJSAFSJFAJS.

#### Procedure

The children had to say whether each sentence inspired joy, anger, fear or sadness. Then the experimenter invited the children to say whether this sentence was "a very little" [joyful], "a little" [joyful], "a lot" [joyful] or "extremely" [joyful]. The experimenter checked the response of the children in the appropriate box. To ensure that the children understood the sentences correctly, and to help them in the selection of the most relevant and non-ambiguous items, children were allowed to ask questions during the test.

### Results

To select the sentences with the greatest potential to induce specific emotions, we conducted two analyses. The first analysis is based on the percentage of correct identification of the primary emotion. The data were analyzed through a one-way ANOVA for the 4 grades. The results showed a significant effect for grade, *F*(3, 62) = 3.5, *p* < .03, η^2^_p_ = .14. The post-hoc analysis showed that the grade effect concerned only the Anger emotion, *F*(3, 151) = 5, *p* < .01, η^2^_p_ = .09. Performance regarding correct identification increased linearly with the rise in grades and therefore with age (2^nd^ grade = 54.33% [5.3], 3^rd^ grade = 54.34% [4.49], 4^th^ grade = 60.85% [4.65], 5^th^ grade = 76.46% [4.6]).

On the basis of participants’ comments and questions, some sentences were perceived as ambiguous or as provoking opposing emotional reactions. For instance, the joy-inspiring sentence "Today, you are going to have lunch with your friends at McDonald’s" was suppressed because of reactions such as "I don’t like McDo, may I change the label to XXX?". The sadness-inspiring item "Your mom threw away your favorite game by mistake" was suppressed because of the ambiguity of the provoked emotion (between sadness and anger). As a consequence, we carried out a more qualitative analysis of all the sentences based on the corpus compiled from the children’s answers. This qualitative step led us to remove three sentences for Joy, two sentences for Anger, one sentence for Fear and two sentences for Sadness.

The second analysis concerned the perceived intensity for each sentence. After identifying the emotion evoked by sentences, participants had to rate their intensity (from 1 "a very little" to 4 "extremely"). All sentences with an average of less than 1.5 were considered to have scored too low and were discarded.

Finally, with regard to the quantitative and qualitative analyses, six sentences per emotion were retained to form our material for inducing specific emotions. Table 2 presents the results of the intensity ratings phase.

**Table 2:** Mean Intensity Ratings of the 6 Sentences per Emotion

Joy Induction	*M*
1. It’s your birthday and all your friends have come with a lot of amazing presents.	3.74
2. Mom decided to surprise you by picking you up at school before lunch time. Today, you don’t eat at school, and you go to the cinema to watch your favorite cartoon.	3.66
3. You dreamt that you were playing at bouncing on a giant trampoline. When you woke up, your dad offered to go and buy a trampoline to play with in your garden.	3.48
4. You are on holiday. You go with your mom and dad to the sea. You took your swimsuit to go in the sea and you have your bucket and spade to build sand castles.	3.37
5. It is Saturday, you are not at school. The sun is shining and it is warm outside.	3.23
6. You give the correct answer to a question from your teacher. She congratulates you in front of all your classmates.	2.60
Anger Induction	
1. A pupil stole something from the teacher’s bag. He said it was you.	3.14
2. You helped a friend to do his homework. The teacher congratulated your friend in front of all your classmates but punished you for having let him copy from you.	2.58
3. Your brother stole some candies from a jar. He did not admit it was him. Your parents punished you both.	2.22
4. You hurt yourself badly by falling down. You shout loud to call for help. The activity leader arrives and tells you off because you’re making too much noise.	1.68
5. Your best friend comes to your house to play with you. Your brother shows your friend an amazing present he had for Christmas. They play together and you stay alone.	1.56
6. Your teacher told you off in front of all your classmates for talking with your neighbor. The truth is that it was your neighbor who talked to you and you did not say a word.	1.52
Fear Induction	
1. You come home with your friend and it is dark. Suddenly, you cannot see your friend anymore and you feel a hand that grabs you.	2.69
2. You are climbing a tree to catch an apple. At the top of the tree, you look down and notice how high it is and you cannot get down.	2.49
3. You have a nightmare. A hideous monster is running after you in the forest. You are running fast and you are shouting during your sleep, but you are alone.	2.30
4. It is night. You suddenly hear a weird noise in the house like "creak, creak, creak".	2.25
5. You are at the swimming pool. You get undressed in the changing room and when you want to open the door, you find it’s stuck.	2.24
6. You are riding your bicycle in the neighborhood. You are going down a steep hill when you suddenly notice the brakes don’t work.	2.22
Sadness Induction	
1. Your best friend moves to another city. You will not see him again.	2.98
2. Your father is really disappointed by your behavior. He says he will not trust you again.	2.20
3. Nobody wished you a happy birthday.	2.15
4. Even though you’d been on your best behavior, you did not receive any presents for Christmas.	1.99
5. The cake you’d been baking all day for your mom’s birthday just burnt. You have nothing to offer her.	1.99
6. Hugo, who you really like, said horrible things about you and that he did not want to play with you anymore.	1.85

*Note.* J = Joy; A = Anger; S = Sadness; F = Fear; kept for the following steps based on a four-point scale (1: A Very Little to 4: A Lot).

#### Discussion

The aim of Experiment 2 was to select a pool of day-to-day emotional situations strongly associated with a primary emotion by the children at different ages. The goal of this selection was to provide suitable verbal induction material for school-age children, in compliance with the ethical requirements. Based on children’s comments as well as quantitative analysis, some sentences were discarded because they were not recognized, were too ambiguous or too weak in intensity. There were no differences in the ratings by grade except for anger, with a linear increase in identification performance with increasing grades. Thus, based on the results of Experiment 2 and for each tested emotion, we selected the 6 sentences assessed by the children as the most closely associated with a primary emotion. These sentences were chosen to serve as induction material in Experiment 3.

## Experiment 3

The main objective of Experiment 1 was to ensure the correct identification of emotion-related adjectives by children from the age of 7. Analysis of the two successive sorting tasks indicated that all the adjectives were correctly grouped according to their valence and their degree of arousal. Based on these results, we then adapted the BMIS scale according to the age/grade of the children. For that, we considered the number of emotional items included in the scale. We used only the items that children categorized correctly in terms of valence and associated primary emotion (for example, "content" was correctly categorized by the children in the 2nd grade as a positive item and correctly associated with "Joy". These scales are presented in Annex A. For children in the 2nd grade, the scale consisted of 7 emotional items, 10 emotional items for the 3rd grade, 11 emotional items for the 4th grade and for the children in the 5th grade the scale is composed of 12 items. On the same principle as the existing BMIS (Mayer & Gaschke, 1988), we developed a scale consisting of the presentation of adjectives in a row, and of a self-rated scale in four points ("*Beaucoup*/A lot", "Un peu/A little", "*Pas trop*/Not very" and "*Pas du tout*/Not at all") in columns.

Based on the results of Experiment 2, we then selected sentences to induce the four studied emotions in children. The induction procedure was inspired by Mayer et al. (1995) to induce fear, anger, happiness and sadness through imagined situations (but without music).

In this third experiment, we induced emotions in children from 7 to 11 years old. Before and after induction, children were asked to rate their emotions on our adapted scale. We expected our tool to find different scores in the assessments of emotional states, depending on the induction.

### Method

#### Participants

The participants were 102 pupils recruited from four grade-levels at an elementary school in Toulouse (France). The distribution was as follows: 2^nd^ grade (24 children, *M* = 7.62 years, *SD* = 0.47 years), 3^rd^ grade (27 children, *M* = 8.61 years, *SD* = 0.41 years), 4^th^ grade (26 children, *M* = 9.90 years, *SD* = 0.52 years) and 5^th^ grade (25 children, *M* = 11.03 years, *SD* = 0.61 years).

#### Ethical Clearance

The same as in Experiment 1.

#### Materials and Design

*BMIS-C (Brief Mood Introspection Scale for Children)*. Based on Experiment 1, four self-rating questionnaires were drawn up and adapted, one for each age group from 7 to 11 (*cf*. Appendix). Each adjective was followed by a series of answer icons: "*Beaucoup*/A lot" (represented by a green + sign 2 x 2 cm), "*Un peu*/A little" (represented by a green + sign 1.5 x 1.5 cm), "*Pas trop*/Not very" (represented by a red – sign 1.5 x 1.5 cm) and "*Pas du tout*/Not at all" (represented by a red – sign 2 x 2 cm). The questionnaire was analyzed by assigning 1 point for the answer "Not at all", 2 points for the answer "Not very", 3 points for the answer "A little" and 4 points for the answer "A lot".

*Induction material by imagination*. On the basis of the results in Experiment 2, four lists of sentences were constructed. Each list contained six sentences that reflected one of the four following emotions: Joy, Anger, Sadness and Fear (see Table 2).

#### Procedure

The induction procedure was collective and carried out with sub-groups of 5 children. The children were in a calm room, each child separated from the others by a certain distance to avoid any communication between them. The experimenter and the teacher were in the room (to comply with the deontological criteria for research in the school environment). Instructions were given orally and were not written on the questionnaires in order to limit the cognitive load for the children. However, during oral instructions, the experimenter wrote on the chalkboard an example using an adjective ("*curieux*/curious") absent from the questionnaire and not reflecting any of the targeted emotions of the study. The instructions were as follows: "I’m giving you a questionnaire with words written one under the other. For each word, I’d like you to circle the symbol that corresponds best to how you feel right now. For example, if we choose the word 'curious', do you feel curious at this moment? If you feel very curious, circle the biggest green + symbol. If you feel a little curious, circle the smallest green + symbol. If you do not feel particularly curious, circle the smallest red - symbol. If you are not curious at all, circle the biggest red - symbol. If there is a word you do not understand, never mind, go on to the next word." After making sure that the children understood the instructions, the experimenter gave them the questionnaires. The children were allowed to take as long as they needed to complete them. Then the eight induction sentences were presented on a white screen, via a video projector. A PowerPoint slideshow was used to present the sentences written in Arial, 18. Sentences were presented one after the other at intervals of 60 seconds. Children were asked to read the sentences and think about them. After this reading and thinking task, children immediately rated their state on the questionnaire (adapted to their age). The induction was followed by a debriefing period that lasted 15 minutes during which the experimenter explained the purpose of the experiment and invited children to explain how they felt and ask any questions they wished.

### Results

The data were analyzed with Sign tests for each of the four types of induction (Joy, Anger, Fear and Sadness), and the four school grades (2^nd^ grade, 3^rd^ grade, 4^th^ grade, 5^th^ grade). The Sign test compared the emotional states of the children at the beginning and end of the experiment. When the delta score (score after - score before) was positive, the emotion was considered to have increased. When it was negative, the studied emotion was considered to have decreased.

#### Induction of Joy

The results showed an effect of the type of induction characterized by an increase of joy in all the children after the induction (all *p*’s < .05). For the children from the first grade, the joy induction did not affect the other emotions. However, the joy induction slightly increased negative emotions of sadness and anger for the children of the second and third grade, as well as sadness for the fourth grade (*cf*. Table 3).

**Table 3:** Evolution of the Emotional State Before and After the Joy Induction, According to the Student’s Grade and the Induced Emotion

Grade	Joy		Anger		Sadness		Fear	
	BMIS-C Before	BMIS-C Delta	BMIS-C Before	BMIS-C Delta	BMIS-C Before	BMIS-C Delta	BMIS-C Before	BMIS-C Delta
1^st^ grade	7.5	3.5*	3	0				
2^nd^ grade	6	5*	4	0*	2	1*	1	0
3^rd^ grade	6.5	4.5*	4	1*	4	1*	2.5	1*
4^th^ grade	7	4*	4	1*	4	1*	4	1*

*Note.* BMIS-C Before = Actual Score of Children in the Bmis-C; Bmis-C Delta = Evolution (BMIS-C Score After – BMIS-C Before).

*p < .05.

#### Anger Induction

Results showed a decrease in joy and an increase in anger for all participants in the experiment, whatever their grade (all *p’*s <.05). It is worth noticing that anger induction did not influence the fear and sadness emotions, except for the older children, who demonstrated an increase in sadness after anger induction (*p* < .04, *cf.* Table 4).

**Table 4:** Evolution of the Emotional State Before and After Anger Induction, According to the Student’s Grade and the Induced Emotion

Grade	Joy		Anger		Sadness		Fear	
	BMIS-C Before	BMIS-C Delta	BMIS-C Before	BMIS-C Delta	BMIS-C Before	BMIS-C Delta	BMIS-C Before	BMIS-C Delta
1^st^ grade	10	-5*	3	4*				
2^nd^ grade	10	-5*	4	6*	3	0	1	0
3^rd^ grade	10	-6*	5	5*	5	0	3	0
4^th^ grade	9.5	-4.5*	4	6*	5	0*	4	1

*Note.* BMIS-C Before = Actual Score of Children in the BMIS-C; BMIS-C Delta = Evolution (BMIS-C score After – BMIS-C Before).

*p < .05.

#### Fear Induction

Results showed a significant decrease in joy, and an increase in anger after the induction of fear, whatever the grade of participants (all *p’s* <.05). In addition, only the older participants in the experiment showed an increase in sadness after induction. Finally, and more surprisingly, none of the children demonstrated any variation in fear after they were induced with fear sentences (*cf*. Table 5). Experiment 1 revealed that fear is confounded with the other negative valence emotions, which might explain why the induction of fear ultimately increases anger and sadness but has a marginal influence on self-assessment of fear.

**Table 5:** Evolution of the Emotional State Before and After Fear Induction, According to the Student’s Grade and the Induced Emotion

Grade	Joy		Anger		Sadness		Fear	
	BMIS-C Before	BMIS-C Delta	BMIS-C Before	BMIS-C Delta	BMIS-C Before	BMIS-C Delta	BMIS-C Before	BMIS-C Delta
1^st^ grade	8	-3*	3	0.5*				
2^nd^ grade	9	-4*	4	2*	3	1*	1	2*
3^rd^ grade	7	-2*	4	1*	6	0	3	4*
4^th^ grade	9	-3*	4	2*	5	0	5	4*

*Note.* BMIS-C Before = Actual Score of Children in the BMIS-C; BMIS-C Delta is the Evolution (BMIS-C score After – BMISc Before).

*p < .05.

#### Sadness Induction

Sadness induction led to a significant decrease in joy, and a significant increase in sadness, fear and anger for all the grades (all *p’*s < .05, *cf*. Table 6).

**Table 6:** Evolution of the Emotional State Before and After Sadness Induction, According to the Student’s Grade and the Induced Emotion

Grade	Joy		Anger		Sadness		Fear	
	BMIS-C Before	BMIS-C Delta	BMIS-C Before	BMIS-C Delta	BMIS-C Before	BMIS-C Delta	BMIS-C Before	BMIS-C Delta
1^st^ grade	10	-5*	3	1*				
2^nd^ grade	10	-6*	3	3*	3	3.5*	1	1*
3^rd^ grade	10.5	-5.5*	4	1*	4	6*	2	1*
4^th^ grade	10	-5*	4	2*	4	6*	4	1*

*Note.* BMIS-C Before = Actual Score of Children in the BMIS-C; BMIS-C Delta = Evolution (BMIS-C score After – BMIS-C Before).

*p < .05.

## General Discussion

The purpose of the present paper was to determine children’s knowledge of their own emotional state. To this end, three experiments were conducted on children from 7 years old. Experiment 1 showed that the knowledge of emotional vocabulary develops with age. At 7, the only dimension used to distinguish emotions is valence. At 9, the degree of arousal is then also used, allowing children to distinguish between emotions with the same valence, such as fear and sadness. An analysis of the results from Experiment 1 enabled us to select the emotional vocabulary items to be used in the creation of an inspection scale suitable for young children. The second experiment showed that 7-year-old children are able to identify the emotion conveyed by scenarios written in the second person singular. This study was also used to select scenarios for the design of the "induction-by-imagination material" of Experiment 3. The selected scenarios were those inducing the emotion of greatest intensity in Experiment 2. Finally, the last experiment shows that from 7 years old, children can be influenced by emotional induction, showing variation in their emotional state after the reading of emotional scenarios. Analysis also revealed that the variations of their emotional state are clearly compatible with the induced emotion when it is negative, while results are more mitigated when the induced emotion is positive. Induced joy leads to an expected increase in joy, but also a less expected weak increase in anger and sadness. Previous studies on emotional induction conducted with adults already underline this odd phenomenon. Inducing joy is the most difficult to achieve, irrespective of the material used (e.g., Mauss, Tamir, Anderson, & Savino, 2011).

### Effect of the Induction-by-Imagination Procedure on Children’s Emotional State

This paper can be considered as the first to investigate both the questions of (1) the self-assessment of emotional state by children and (2) the influence of emotional induction by imagination on children’s emotional behavior from the age of 7. Previous studies have shown that the investigation of children’s emotional self-assessment is commonly conducted on an external object, with the children having to attribute or identify the emotion conveyed by a face or an animate or inanimate object (e.g., Guarnera, Hichy, Cascioa, & Carrubba, 2015; Kothari, Skuse, Wakefield, & Micali, 2013; Reilly, McIntire, & Bellugi, 1990). This study investigated the capacity of children to feel the emotion conveyed by a scenario, and then to show compatible variation in their own emotional state. It appears that children can feel the emotion conveyed by a scenario within the limits of their own knowledge of what the induced emotion is (Saarni, 2011). Adopting an adult-centered view of emotion involves the assumption that many emotions are distinguished on the basis of their various dimensions (valence, arousal, dominance, etc.). It appears that the understandable emotional world of children, even at age 9, is principally (not to say exclusively) defined by the valence of the emotion. Analysis of Experiment 1 clearly showed how difficult it was for our younger participants (up to age 9) to correctly categorize items such as "grieved", "scared", and "afraid" in separate groups.

### Measuring Emotional Development

Numerous studies have been performed on the development of emotions from very early childhood, and several of them demonstrate that even very small babies are able to distinguish emotions (e.g. Caron, Caron, & MacLean, 1988; Izard & Harris, 1995). The critical elements that could partly explain our result are the fact that (1) the material used in our study is verbal rather than pictural (e.g. Guarnera, Hichy, Cascioa, & Carrubba, 2015), and that (2) we wanted participants to distinguish and identify more than two emotions (e.g. LaBarbera, Izard, Vietze, & Parisi, 1976). Finally, we believe our study contributes significantly to the understanding of normal or ordinary emotional development from the age of 7. It also attempts to provide new insights into the particular difficulties of acquiring "emotional" knowledge. Research in this field could help professionals (teachers, therapists, etc.) adapt the way they interact and work with children of different ages as appropriate for their knowledge of emotional situations and their emotional regulation capacities.

## Appendix

**Table:** Emotional self-assessment questionnaire for children in 2nd grade

1. Happy (*Heureux*)	**+**	+	-	**-**
2. Sad (*Triste*)	**+**	+	-	**-**
3. Annoyed (*Enervé*)	**+**	+	-	**-**
4. Content (*Content*)	**+**	+	-	**-**
5. Angry (*En colère*)	**+**	+	-	**-**
6. Joyful (*Joyeux*)	**+**	+	-	**-**
7. Furious (*Furieux*)	**+**	+	-	**-**

**Table:** Emotional self-assessment questionnaire for children in 3rd grade

1. Happy (*Heureux*)	**+**	+	-	**-**
2. Sad (*Triste*)	**+**	+	-	**-**
3. Annoyed (*Enervé*)	**+**	+	-	**-**
4. Afraid (*Effrayé*)	**+**	+	-	**-**
5. Content (*Content*)	**+**	+	-	**-**
6. Unhappy (*Malheureux*)	**+**	+	-	**-**
7. Angry (*En colère*)	**+**	+	-	**-**
8. Scared (*Apeuré*)	**+**	+	-	**-**
9. Joyful (*Joyeux*)	**+**	+	-	**-**
10. Furious (*Furieux*)	**+**	+	-	**-**

**Table:** Emotional self-assessment questionnaire for children in 4th grade

1. Happy (*Heureux*)	**+**	+	-	**-**
2. Sad (*Triste*)	**+**	+	-	**-**
3. Annoyed (*Enervé*)	**+**	+	-	**-**
4. Afraid (*Effrayé*)	**+**	+	-	**-**
5. Content (*Content*)	**+**	+	-	**-**
6. Unhappy (*Malheureux*)	**+**	+	-	**-**
7. Angry (*En colère*)	**+**	+	-	**-**
8. Scared (*Apeuré*)	**+**	+	-	**-**
9. Joyful (*Joyeux*)	**+**	+	-	**-**
10. Grieved (*Chagriné*)	**+**	+	-	**-**
11. Furious (*Furieux*)	**+**	+	-	**-**

**Table:** Emotional self-assessment questionnaire for children in 5th grade

1. Happy (*Heureux*)	**+**	+	-	**-**
2. Sad (*Triste*)	**+**	+	-	**-**
3. Annoyed (*Enervé*)	**+**	+	-	**-**
4. Afraid (*Effrayé*)	**+**	+	-	**-**
5. Content (*Content*)	**+**	+	-	**-**
6. Unhappy (*Malheureux*)	**+**	+	-	**-**
7. Angry (*En colère*)	**+**	+	-	**-**
8. Scared (*Apeuré*)	**+**	+	-	**-**
9. Joyful (*Joyeux*)	**+**	+	-	**-**
10. Grieved (*Chagriné*)	**+**	+	-	**-**
11. Furious (*Furieux*)	**+**	+	-	**-**
12. Worried (*Inquiet*)	**+**	+	-	**-**
